# Supplementary material for: Evaluation of in silico algorithms for use with ACMG/AMP clinical variant interpretation guidelines
Source: Genome Biol. 2017 Nov 28;18:225. doi: 10.1186/s13059-017-1353-5 (PMC5704597; doi:10.1186/s13059-017-1353-5)
Supplement: Supplementary file 2 — Concordance among predictions of 18 algorithms for 8386 variants in ClinVar for which predictions were available from all 18 algorithms. Figure S2. Variability in performance of algorithms shown in each panel across all analyzed datasets. Figure S3. Performance analysis of algorithms for the indicated datasets. (PPTX 87 kb) [file 13059_2017_1353_MOESM2_ESM.pptx]

## Slide 1
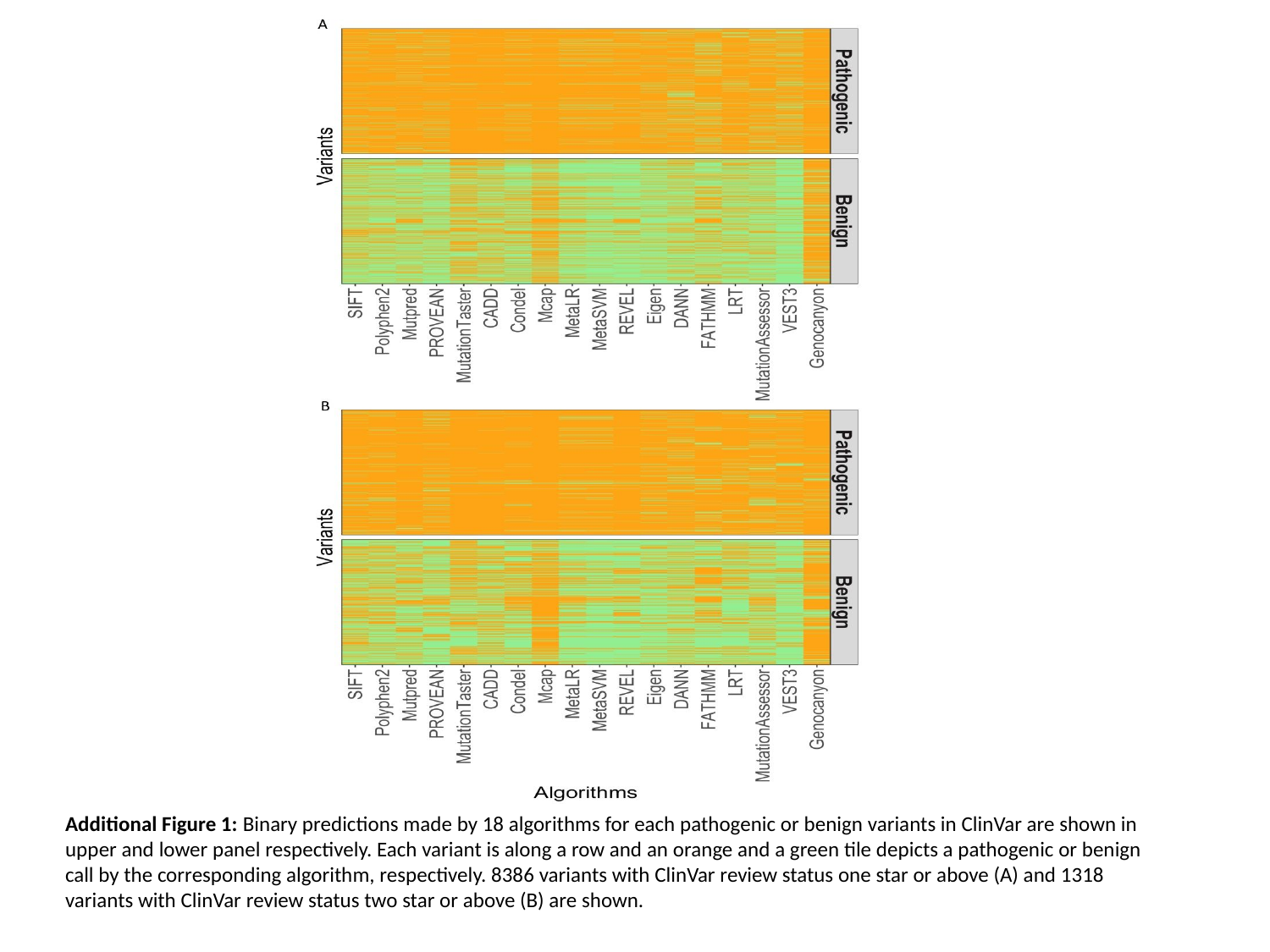

Additional Figure 1: Binary predictions made by 18 algorithms for each pathogenic or benign variants in ClinVar are shown in upper and lower panel respectively. Each variant is along a row and an orange and a green tile depicts a pathogenic or benign call by the corresponding algorithm, respectively. 8386 variants with ClinVar review status one star or above (A) and 1318 variants with ClinVar review status two star or above (B) are shown.

## Slide 2
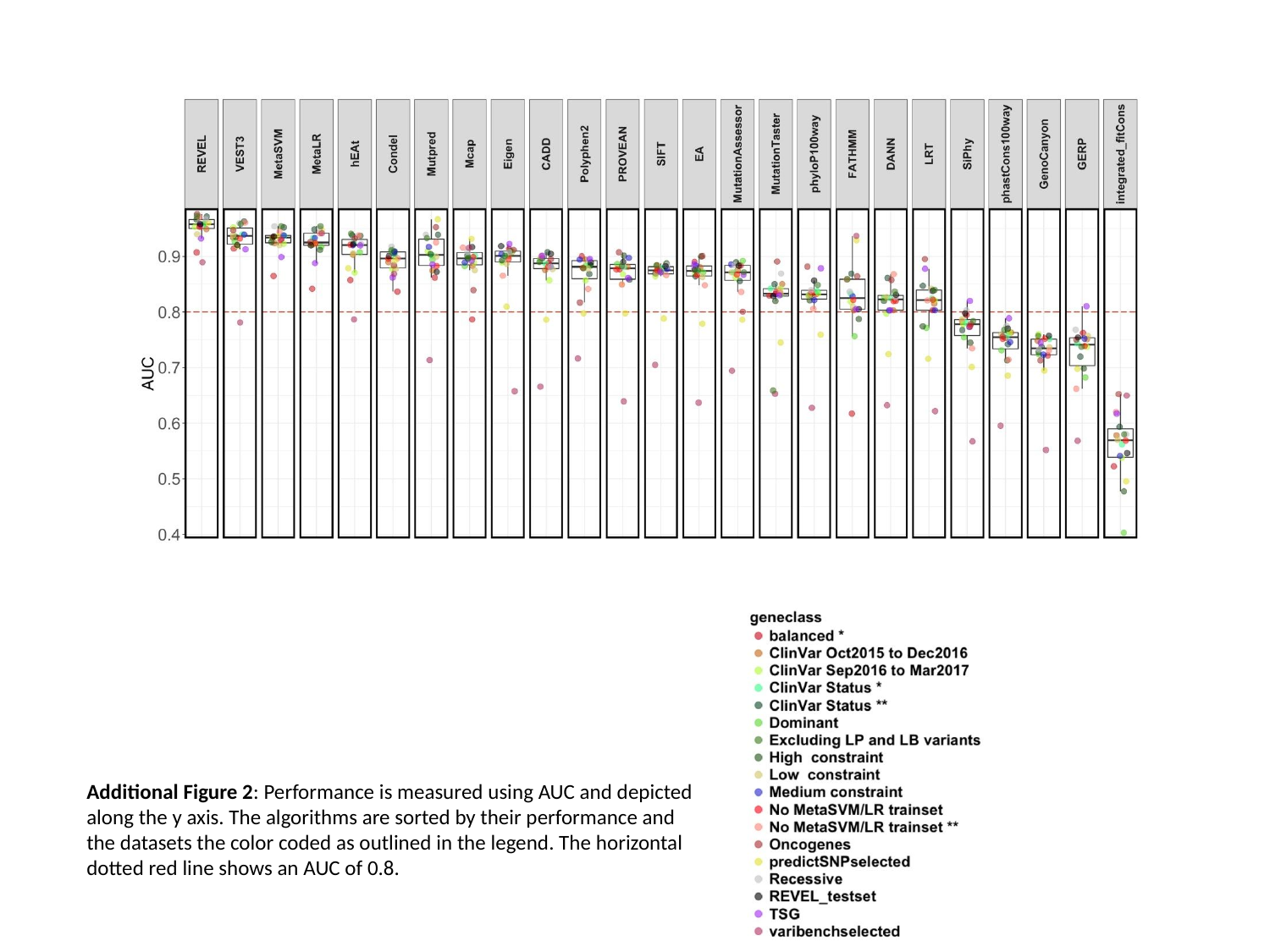

Additional Figure 2: Performance is measured using AUC and depicted along the y axis. The algorithms are sorted by their performance and the datasets the color coded as outlined in the legend. The horizontal dotted red line shows an AUC of 0.8.

## Slide 3
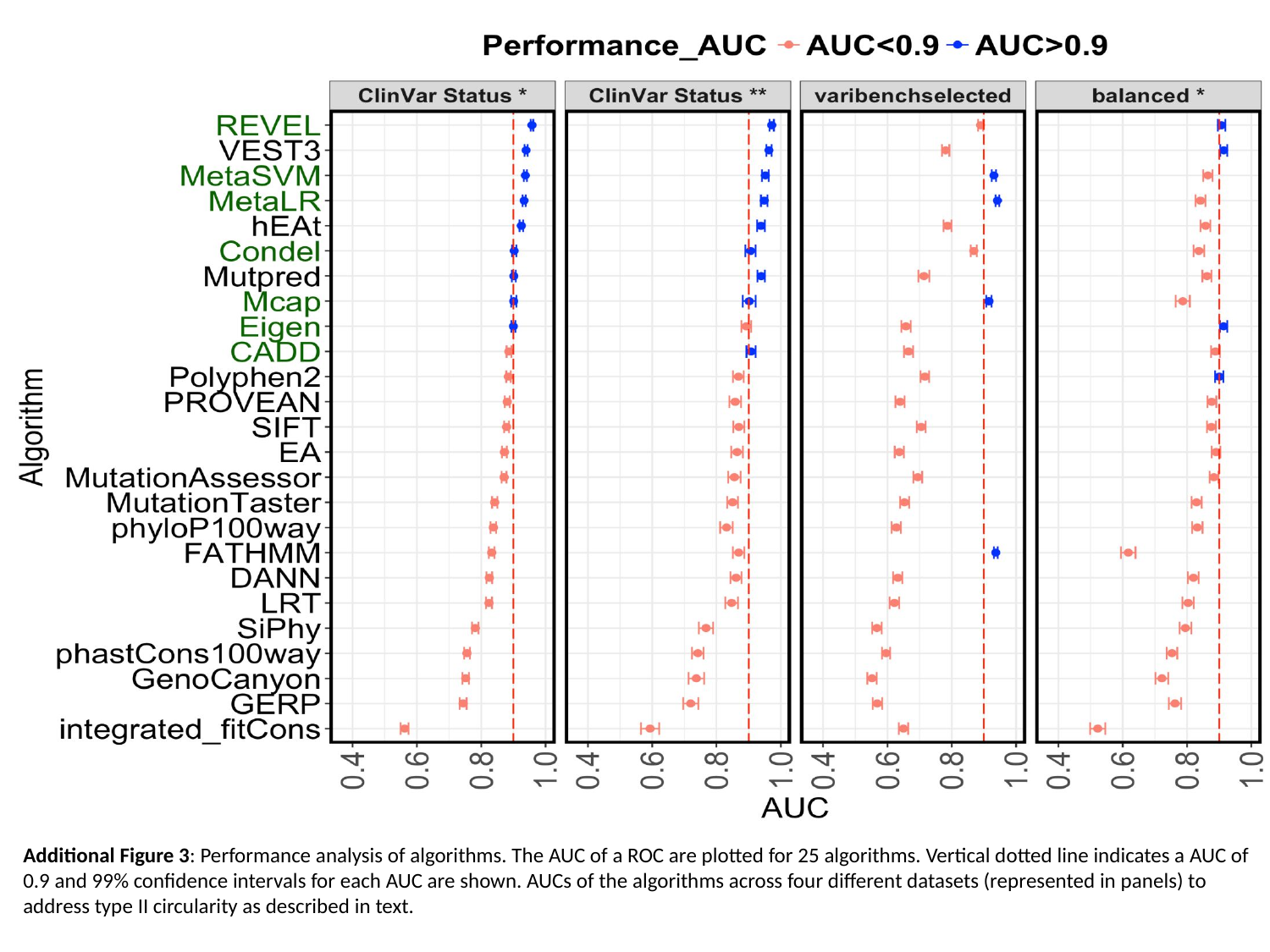

Additional Figure 3: Performance analysis of algorithms. The AUC of a ROC are plotted for 25 algorithms. Vertical dotted line indicates a AUC of 0.9 and 99% confidence intervals for each AUC are shown. AUCs of the algorithms across four different datasets (represented in panels) to address type II circularity as described in text.
